# HTLV-1 genetic diversity of 52 complete sequences from 14 African countries reveals novel variants and a lack of typical P12/P8 and P30 accessory proteins in HTLV-1b, d, and f genotypes

**DOI:** 10.1080/22221751.2026.2651463

**Published:** 2026-03-25

**Authors:** Olivier Cassar, Délia Doreen Djuicy, Giovanni Begliomini, Jill-Léa Ramassamy, Eldridge Fredricksen Oloumbou, Augustin Mouinga-Ondeme, Richard Njouom, Ambroise Marcais, Emilie Deruelle, Olivier Hermine, Vincente Soriano, Carmen de Mendoza, Graham Taylor, Philippe V. Afonso, Antoine Gessain

**Affiliations:** a Institut Pasteur, Unité Épidémiologie and Physiopathologie des Virus Oncogènes, Université Paris-Cité, Paris, France; b Virology Service, Centre Pasteur of Cameroon, Yaoundé, Cameroon; c Centre International de Recherche Médicales de Franceville, Franceville, Gabon; d Necker University Hospital, Paris, France; e UNIR Health Sciences School & Medical Center, Madrid, Spain; f Puerta de Hierro University Hospital & Research Institute, Madrid, Spain; g Imperial College London, London, UK

**Keywords:** HTLV, STLV, Africa, molecular epidemiology, accessory proteins

## Abstract

Central Africa is the largest region of human T-cell Leukaemia virus (HTLV-1) endemicity with several million people estimated to be infected. Based on the study of the LTR region, it is also the region with the highest HTLV-1 diversity, with the presence of genotypes a-b and d-g. However, complete genomic sequences are still lacking for Central African genotypes. Here, we report the first large collection of complete HTLV-1 sequences for genotypes b, d and f from Central Africa and neighbouring countries. We identified substantial diversity within the HTLV-1b genotype, including a newly defined clade that we designated HTLV-1b-del. It mainly comprises strains from the Democratic Republic of the Congo (COD) and neighbouring countries and is characterized by a distinctive 12-bp-long deletion. We also generated the complete sequence of the STLV-1 strain from *Allenopithecus nigroviridis* from the COD. This strain belongs to the PTLV-1b genotype and carries a 12-bp duplication in the pX region. Lastly, we found that, except for HTLV-1a strains, HTLV-1 genomes generally lack open reading frames encoding the canonical accessory protein P12; instead, they encode either shorter versions of the protein or an ORF lacking a start ATG codon. This work substantially expands the genomic landscape of HTLV-1 in Central Africa and provides a critical resource for understanding viral diversity.

## Introduction

The human T-lymphotropic virus type 1, also known as human T-cell Leukaemia virus (HTLV-1), is the etiological agent of two major diseases with severe clinical outcomes: the lymphoproliferative malignancy adult T-cell leukaemia/lymphoma (ATL) and the neurodegenerative disorder tropical spastic paraparesis/HTLV-1–associated myelopathy (TSP/HAM). The virus is also implicated in several inflammatory conditions, including infective dermatitis, myositis, and uveitis [1,2].

It is estimated that at least 5 to 10 million people are infected with HTLV-1 worldwide [3]. Although the virus is distributed across the globe, its prevalence is concentrated in specific high-endemic regions, referred to as foci. Among these, Central Africa represents the largest HTLV-1 endemic region, accounting for roughly one-third of all infected individuals [4].

HTLV-1 is characterized by remarkable genetic stability, primarily resulting from the clonal expansion of infected cells [5]. Based largely on partial *env* gene and LTR sequences, seven major molecular genotypes have been identified, most of which are restricted to specific geographic regions [6,7].

The Cosmopolitan a-genotype includes several subgroups: the Transcontinental (a-TC) subgroup (the most disseminated strain in the world), as well as geographically localized subgroups such as Japan (a-JPN), North Africa (a-NA), West Africa (a-WA), and Senegal (a-Sen). The remaining genotypes consist of the Melanesian/Australian c-genotype and five additional genotypes found mainly in Central Africa (b and d-g), with genotype b being the most prevalent.

Complete genome sequences are still lacking for most Central African HTLV-1 genotypes. To date, only two complete sequences exist for the b-genotype, one from Brazil – SF26 [8] and one originating from Africa – EL [9], and no other full-length sequences have been reported for HTLV genotypes d-g.

STLV-1, the simian counterpart of HTLV-1, can be transmitted to humans through contacts with infected body fluids [10,11]. In Africa, STLV-1 can be found in various non-human primate (NHP) species, including chimpanzees, gorillas, baboons, and mandrills, or African green monkeys [4]. However, only a limited number of complete STLV-1 genome sequences from African NHPs are currently available.

Viral propagation and persistence of the HTLV-1a genotype have been linked to the activity of accessory proteins (P12/P8, P13, and P30) [12]. Although these proteins play key roles in pathogenesis in HTLV-1a, *in silico* analyses suggest that the corresponding ORFs are often altered (either lacking the start ATG codon or splicing sites, or encoding shorter protein versions) [13,14]. In the case of HTLV-1c, the lack of p12/P8 ORFs appears to be compensated by alternative splicing leading to the production of the P16 protein [15,16].

In this study, we generated a large collection of complete HTLV-1a, b, d, and f genomes from Central Africa, neighbouring countries, and the French département of la Réunion. Though this work, we identified substantial diversity within the HTLV-1b genotype, including a newly defined clade that we designated HTLV-1b-del, with a distinctive deletion that results in the absence of the ORFs encoding the canonical P12/P8 and P30 accessory proteins. We generated the first complete genome sequences for HTLV-1 genotype d and f. We also identified a divergent strain from the Central African Republic (CAR), PH511. Finally, we also generated a complete STLV-1b sequence from the Democratic Republic of the Congo.

## Material and methods

### Sample collection and ethics statement

Samples from Gabon [17] and Cameroon [18] (except H23 and H24) were collected in the context of epidemiological studies. Details regarding ethical approvals are provided in the corresponding references.

Most samples were obtained from hospitals in France, the United Kingdom, and Spain, where HTLV-1-infected patients are routinely diagnosed and followed. In France, the Biomedical Research Program was approved by the *Comité de Protection des Personnes* Ile-de-France II, Paris (2012-10-04 SC) (strains IYA, PH1614, PH1516, PH1517, PH1617, PH1636, PH1812).

In the United Kingdom, samples (from Nigeria, Uganda, Swaziland, Zambia, and Zimbabwe) were provided by the Communicable Diseases Group Tissue Bank (ethics approval reference 15/SC/0089). The sample collected in Spain (2057) was obtained from the Spanish HTLV Network repository (ethics approval reference PI/185-21) [19].

The remaining strains were part of a biological collection, declared to the *Ministère de l’Enseignement Supérieur et de la Recherche* (2010 DC-1197).

All participants provided written informed consent prior to inclusion.

We also included in this study the STLV sequences from two monkeys (*Allenopithecus nigroviridis*) originating from the Democratic Republic of the Congo [20].

### PCR amplification and generation of PTLV-1 genomes

DNA samples were amplified using four overlapping PCR assays (F1-F4) covering the complete HTLV-1 genome. These assays targeted the LTR-*gag* (F1), *gag*-*pro* (F2), *pol*-*env* (F3), and pX-LTR (F4) regions, as previously described [7,21]. Details of the sequencing procedures are provided in the supplementary material and methods.

### Phylogenetic analysis

Sequence alignments were performed using SeaView 5 (v5.0.4) [22].

The most appropriate nucleotide substitution model was selected using the Modeltest v3.6 program in combination with PAUP (v4.0) [23].

Phylogenetic trees were inferred using the Phylogenetic Maximum Likelihood (PhyML) or parsimony using SeaView5. Regarding partitioning, both NNI (Nearest neighbour interchange) and SPR (Subtree pruning and regrafting) rearrangements were tested. Clade support was evaluated using the approximate likelihood-ratio test (aLRT).

Topologies were confirmed with the PhyML method using IQ-Tree and ultrafast bootstrap [24].

### HTLV-1 recombinant search

Recombinant screening and breakpoint detection were performed as previously described [7]. Boot scanning analyses were conducted using SimPlot v3.5.1.

For long terminal repeat (LTR) analysis, a window size of 200-bp with a step size of 20-bp was applied. For analysis of the complete genome, a window size of 800-bp with a step size of 80-bp was used. The use of different window sizes reflects the lower genetic variability observed in certain genomic regions.

For each window, bootstrap values between the query and references were calculated according to the Kimura two-parameter model with 1000 replicates.

### *In silico* analysis of viral ORFs

The various putative viral mRNAs were identified by comparison with previously described open reading frames and splicing donor and acceptor sites of the ATK-1 reference strain [25,26].

## Results

### Generation of HTLV-1 complete genomes and their distribution in Africa

**Figure 1.**
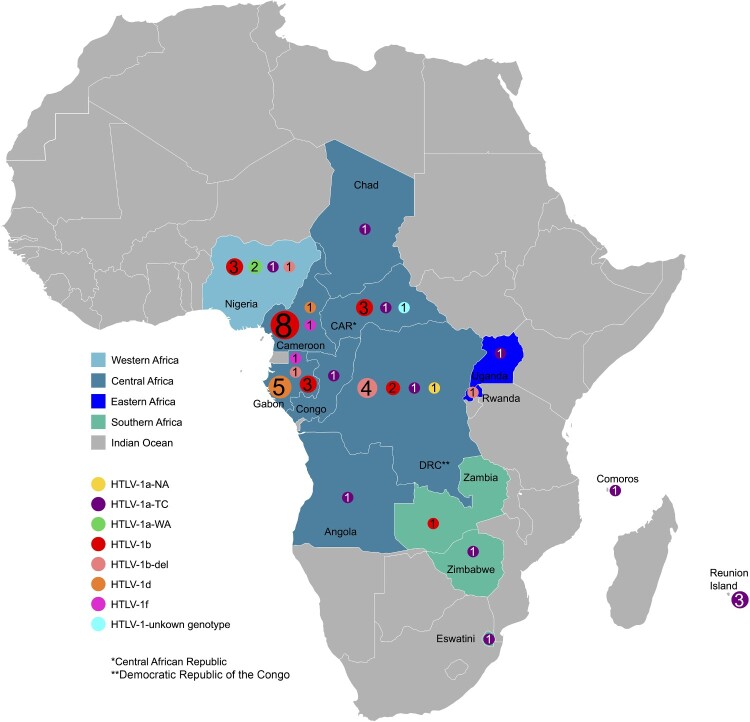
Geographical distribution of HTLV-1 genotypes identified in this study. Notes: The donors originated from 14 countries across Africa and the Indian Ocean, or the French department of La Réunion. TC, Transcontinental; NA, North African; WA, West African.

We generated complete HTLV-1 genomic sequences from 52 infected individuals of various clinical status. The infected persons originated from various regions of Africa (Figure 1, Table 1). Thirty-four of these samples were previously sequenced for the LTR region and analysed in earlier studies [7,27,28].

**Table 1.** Epidemiological data and clinical status of the 52 HTLV-1-infected donors.

Region of origin	Country (ISO Code)	Strain ID	Age (y)	Gender	HTLV-1 Clinical status	HTLV-1 subtype	Accession Number
West Africa	Federal Republic of Nigeria (NGA)	Nig23	46	F	HAC	HTLV-1b	PZ133388
		Nig64	72	F	TSP/HAM	HTLV-1a-WA	PZ133389
		Nig21	51	F	TSP/HAM	HTLV-1a-TC	PZ133390
		HFS	34	M	HAC	HTLV-1b	PZ133391
		HGS	64	F	HAC	HTLV-1b-del?	PZ133392
		HIK	41	F	HAC	HTLV-1a-WA	PZ133393
		2057	49	F	ATL	HTLV-1b	PZ133394
Central Africa	Gabon (GAB)	007-OI	25	M	HAC	HTLV-1b	PZ133395
		184-NG	77	F	HAC	HTLV-1b	PZ133396
		358-NG	45	F	HAC	HTLV-1d	PZ133397
		WN-261	45	M	HAC	HTLV-1d	PZ133398
		195-NG	29	M	HAC	HTLV-1f	PZ133399
		IYA	49	F	ATL	HTLV-1b	PZ133400
		WN-218	Adult	F	HAC	HTLV-1d	PZ133401
		WN-196	Adult	F	HAC	HTLV-1d	PZ133402
		057-OL	54	M	HAC	HTLV-1d	PZ133403
		275-HO	48	M	HAC	HTLV-1b-del	PZ133404
	Central African Republic (CAF)	PH511	46	F	TSP/HAM	HTLV-1	PZ133405
		PH1812	50	F	ATL Lymphoma	HTLV-1a-TC	PZ133406
		12503	60	M	HAC	HTLV-1b	PZ133407
		12504	50	F	HAC	HTLV-1b	PZ133408
		12505	14	M	HAC	HTLV-1b	PZ133409
	Angola (AGO)	PH549	32	M	ATL	HTLV-1a-TC	PZ133410
	Democratic Republic of the Congo (COD)	PH1250	44	F	HAC	HTLV-1b-del	PZ133411
		PH1813	64	M	TSP/HAM	HTLV-1b-del	PZ133412
		PH648	54	F	TSP/HAM	HTLV-1a-NA	PZ133413
		LUZ.N	53	F	ATL ACute	HTLV-1a-TC	PZ133414
		PH1636	50	F	TSP/HAM	HTLV-1b-del	PZ133415
		PH660	17	F	HAC	HTLV-1b	PZ133416
		418	25	M	HAC	HTLV-1b-del	PZ133417
		PH1614	59	M	HAC	HTLV-1b	PZ133418
	Cameroon (CMR)	BAK56	65	M	HAC	HTLV-1b	PZ133419
		BAK133	51	M	HAC	HTLV-1b	PZ133420
		BAK186	45	M	HAC	HTLV-1b	PZ133421
		BAD22	22	M	HAC	HTLV-1f	PZ133422
		BAK40	35	M	HAC	HTLV-1b	PZ133423
		H23	44	M	HAC	HTLV-1d	PZ133424
		H24	29	F	HAC	HTLV-1b	PZ133425
		Pyl-42	24	F	HAC	HTLV-1b	PZ133426
		Pyl-114	28	M	HAC	HTLV-1b	PZ133427
		BAD447	56	M	HAC	HTLV-1b	PZ133428
	Republic of the Congo (COG)	PH63	32	M	TSP/HAM	HTLV-1a-TC	PZ133429
	Chad (TCD)	PH996	38	F	HAC	HTLV-1a-TC	PZ133430
East Africa	Uganda (UGA)	HDK	46	F	HAC	HTLV-1a-TC	PZ133431
	Rwanda (RWA)	PH1617	57	M	HAC	HTLV-1b-del	PZ133432
South Africa	Eswatini (SWZ)	TCY	52	F	TSP/HAM	HTLV-1a-TC	PZ133433
	Zambia (ZMB)	TDS	62	F	TSP/HAM	HTLV-1b	PZ133434
	Zimbabwe (ZWE)	3TDJ	55	F	TSP/HAM	HTLV-1a-TC	PZ133435
Indian Ocean	Comoros (COM)	PH1361	52	F	TSP/HAM	HTLV-1a-TC	PZ133436
	France, La Réunion (REU)	PH1516	96	F	HAC	HTLV-1a-TC	PZ133437
		PH1517	57	F	ATL	HTLV-1a-TC	PZ133438
		Vid.D	59	F	TSP/HAM	HTLV-1a-TC	PZ133439

Notes: Donors originated from multiple countries in Central Africa, neighbouring countries, and the French department of La Réunion. Country identification follows the ISO 3166 standard, except for La Réunion, which is designated as REU. Each donor was assigned a unique StrainID, and the corresponding accession number on GenBank is provided. Genotypes are indicated, and HTLV-1a subclades are designated as TC (Transcontinental), WA (West Africa), and NA (North Africa). Donors were either asymptomatic carriers (HAC) or patients diagnosed with Adult T-cell Leukaemia (ATL) or Tropical Spastic Paraparesis / HTLV-1 associated myelopathy (TSP/HAM).

The size of most complete genomes ranged from 9,035 to 9,037 nucleotides in length. The 2 HTLV-1d strains from Gabon were slightly smaller, i.e. 9,018 and 9,019-nt-long, respectively.

Among the 52 samples analysed, three familial clusters were identified:

- A couple living in Cameroon (Pyl-42 and Pyl-114) was infected with a strain of genotype b, exhibiting a single nucleotide difference.
- A family of three members living in the Central African Republic comprised a couple (12,503–12,504) and their son (12,505). The three HTLV-1b strains were very similar (> 99.9% nucleotide identity; 10 nucleotides divergence between the couple, 7 nucleotide divergence between each parent and their son).
- An ATL patient (PH1517) and her mother (PH1516) from La Réunion were infected with HTLV-1a strains that exhibited only a 3-nucleotide difference.

**Figure 2.**
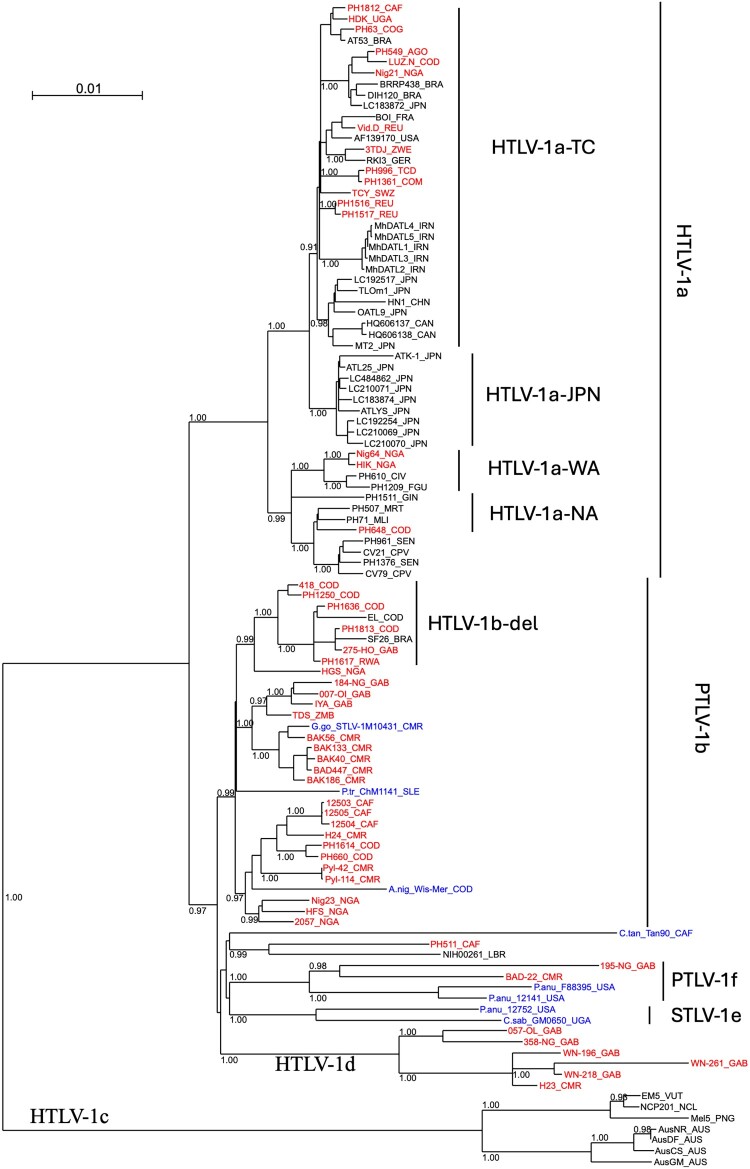
Phylogenetic tree based on the alignment of concatenated gag-pro-pol + env + tax genes. Notes: Phylogenetic analysis is based on a 6915-bp alignment of concatenated gag-pro-pol + env + tax genes, using PhyML in SeaView, with the Tam-Nei model (TN93 + I + G). Rearrangements using NNI and SPR are used. Branch lengths are drawn to scale, with the bar indicating 0.01 nucleotide replacement per site. Numbers on each node indicate the value of the approximate likelihood-ratio test (aLRT), presented as a probability. HTLV-1c genotype is used as outgroup. The newly obtained sequences are presented in red for HTLV-1. STLV-1 strains are presented in blue, and the first letters correspond to the NHP species. Countries of origin are indicated by their three-letter code (ISO 3166). TC, Transcontinental; JPN, Japan; WA, West African, NA, North Africa. STLV, PTLV and HTLV mean simian, primate and human T-lymphotropic virus, respectively.

**Figure 3.**
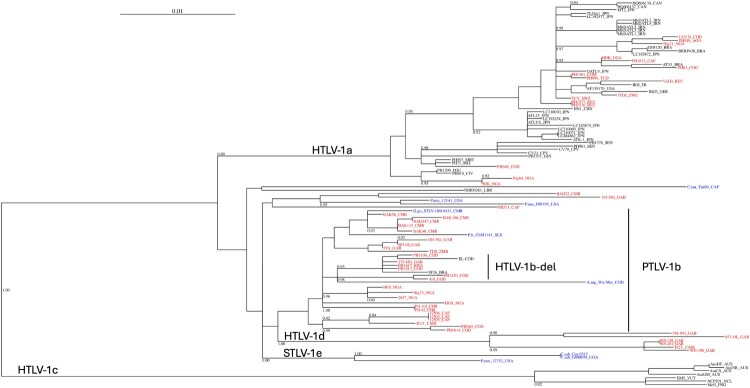
Phylogenetic tree of PTLV-1 based on the alignment of complete LTR sequences. Notes: Phylogenetic analysis is based on a 774-bp alignment of complete LTR, using PhyML in SeaView, with the GTR + I + G model. Rearrangements using NNI and SPR are used. Branch lengths are drawn to scale, with the bar indicating 0.01 nucleotide replacement per site. Numbers on each node indicate the value of the approximate likelihood-ratio test (aLRT), presented as a probability. HTLV-1c genotype is used as outgroup. Countries of origin are indicated by their three-letter code (ISO 3166). STLV, PTLV and HTLV mean simian, primate and human T-lymphotropic virus, respectively.

Phylogenetic analyses on gag-pro-pol + env + tax concatenated sequences indicate that 27 strains belong to HTLV-1b, 6 to HTLV-1d, 2 to HTLV-1f, and 16 to HTLV-1a (Figure 2, Figures S2–3, Table 1). Importantly, phylogenetic tree topology was confirmed when analysing LTR sequences – either complete LTR (Figure 3) or devoid of any ambiguous nucleotide position in the alignment (Figure S4).

Interestingly, we found that highly supported and geographically related clades seemed to emerge within the HTLV-1b genotype. For instance, 3 out of 4 HTLV-1b strains from Nigeria clustered together. Similarly, 5 out of 8 HTLV-1b strains from Cameroon were found in the same clade. However, it remains unclear whether these groups are supported by a strong phylogenetic signal or instead result from the reduced number of strains and a recruitment bias.

In contrast, HTLV-1d and f exhibited long branches.

Furthermore, one sequence from CAF (PH511) was found divergent. Although present on a long branch, its closest known strain is NIH00261, which was isolated from a patient from Liberia [29]. However, the position varies slightly depending on phylogenetic analysis (Figure 2 vs Figure 3). PH511 and NIH00261 show only 97% identity in concatenated genes and 95.5% identity in the complete LTR. Neither strain could be associated with a previously defined genotype.

We have previously reported that some clades within HTLV-1a genotype (a-NA and a-G-Rec) have originated from recombination [7,27]. We therefore investigated whether a recombination signature could be detected among the HTLV-1b strains, or when using PH511 as the query sequence. No evidence of such recombination was identified. The only strain that seemed to arise from recombination was PH648 from the COD, which belongs to the previously reported recombinant group a-NA.

### Identification of a novel clade within HTLV-1b presenting a 12-nt long deletion, HTLV-1b-del

**Figure 4.**
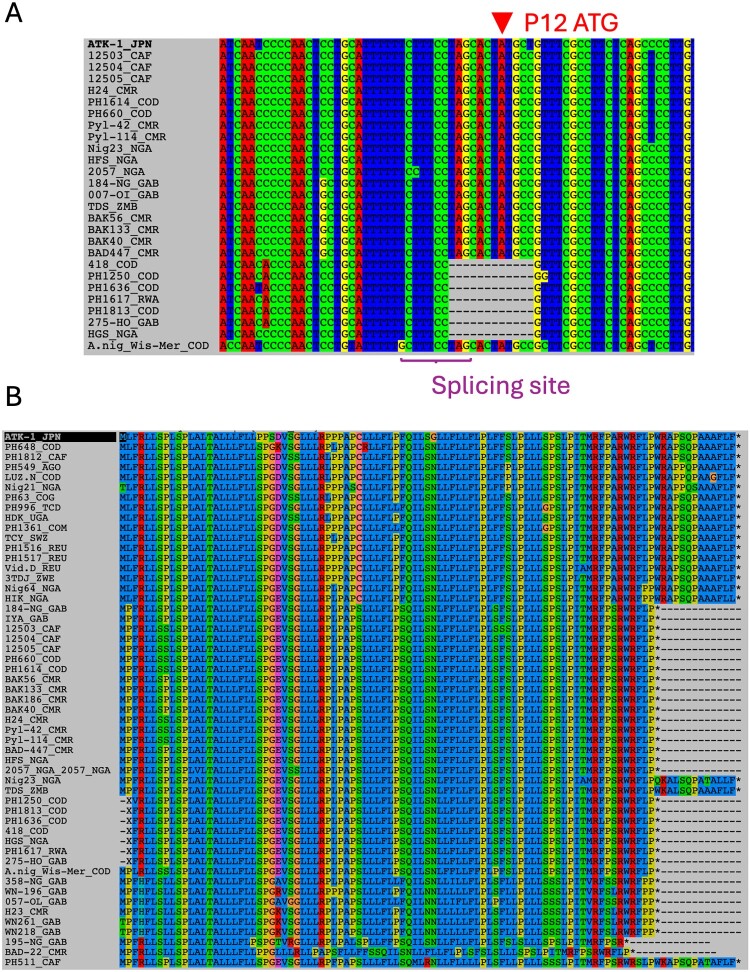
Presentation of accessory proteins of the newly identified HTLV-1b strains. (A) Description of the 12-bp deletion in HTLV-1b-del strains, and consequences on accessory genes. Alignment is observed using Seaview. The newly generated PTLV-1b strains are presented. The initiation codon of P12/P8 and the splicing site for P12/P8 and P30 are absent in HTLV-1b-del. The prototypic ATK-1 strain is used as reference. The ATG position corresponds to nucleotide 6875 on the alignment. (B) Putative sequence corresponding to P12 from the newly generated strains. Strains are organized according to genotypes (see Table 2). Amino-acids are presented using the corresponding 1 letter code.

**Figure 5.**
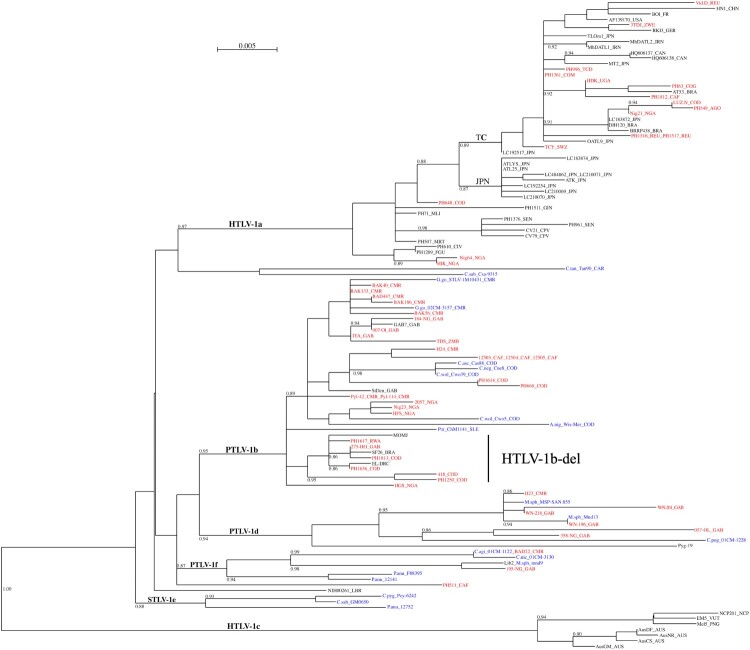
Phylogenetic tree of PTLV-1 based on the alignment of partial LTR sequences. Notes: Phylogenetic analysis is based on a 544-bp alignment of partial LTR, using PhyML in SeaView, with the GTR + I + G model. Rearrangements using NNI and SPR are used. Branch lengths are drawn to scale, with the bar indicating 0.005 nucleotide replacement per site. Numbers on each node indicate the value of the approximate likelihood-ratio test (aLRT), presented as a probability. HTLV-1c genotype is used as outgroup. The newly obtained sequences are presented in red for HTLV-1. STLV-1 strains are presented in blue, and the first letters correspond to the NHP species. TC, Transcontinental; JPN, Japan; STLV, PTLV and HTLV mean simian, primate and human T-lymphotropic virus, respectively.

Within the HTLV-1b genotype, we identified a monophyletic clade that we named HTLV-1b-del (Figures 2 and 3, Figure 5, and Figures S2-4). This clade comprises sequences sharing a 12-nucleotide deletion located at positions 6,828–6,839 relative to the ATK-1 reference strain (Figure 4(A)). The clade comprises 4 strains from the COD, as well as strains from Gabon and Rwanda. The clade also contains the previously reported complete HTLV-1b strains SF26 from Brazil and EL from the COD [8,9]. The monophyletic group suggests that these viruses derive from a common ancestral strain carrying this deletion.

A strain from Nigeria (HGS) exhibited a similar deletion; however, in phylogenetic analysis it frequently appears at the base of the HTLV-1b-del clade (Figure 2 and Figures S2-4) and, in some cases, forms a separate lineage (Figure 3).

To further support the existence of this recurrent deletion site, we generated partial sequences spanning the deleted region from 11 HTLV-1b–infected individuals originating from the COD, as insufficient high-quality DNA prevented the generation of complete genomes. Among these 11 strains, two carried the deletion at the same position observed in the HTLV-1b-del clade (data not shown). Overall, 6 out of 17 HTLV-1b strains from COD (35%) belonged to this subclade.

The monophyletic nature of the group and the consistency in the deletion site suggest that the virus is viable and has been transmitted among humans.

### Absence of accessory ORFs encoding P12/P8 and P30 in HTLV-1b-del

Several studies have highlighted the importance of accessory ORFs – encoding P12/P8, P13, and P30 – in viral persistence and propagation [12]. However, the functional relevance of such proteins remains debated [13,14,30].

We performed a similar analysis on the newly generated genomes looking for the presence of the viral genes (Table 2). In HTLV-1a strains (except strain Nig21 from Nigeria, derived from a patient with TSP/HAM), the canonical accessory ORFs remained intact.

**Table 2.** *In silico* analysis of the ORFs encoded by newly reported PTLV-1 strains.

Strain ID	Country	HTLV-1 subtype	Gag	Pol	Env	Tax	Hbz	P12	P13	P30	Clinical status
PH648	COD	HTLV-1a-NA						99 AA			TSP/HAM
PH1812	CAF	HTLV-1a-TC						99 AA			ATL
PH549	AGO	HTLV-1a-TC									ATL
LUZ.N	COD	HTLV-1a-TC									ATL
Nig21	NGA	HTLV-1a-TC						no ATG	elongated ORF		TSP/HAM
PH63	COG	HTLV-1a-TC						99 AA			TSP/HAM
PH996	TCD	HTLV-1a-TC									HAC
HDK	UGA	HTLV-1a-TC									HAC
PH1361	COM	HTLV-1a-TC									TSP/HAM
TCY	SWZ	HTLV-1a-TC									TSP/HAM
PH1516	REU	HTLV-1a-TC									HAC
PH1517	REU	HTLV-1a-TC									HAC
Vid.D	REU	HTLV-1a-TC									TSP/HAM
3TDJ	ZWE	HTLV-1a-TC									TSP/HAM
Nig64	NGA	HTLV-1a-WA						99 AA			TSP/HAM
HIK	NGA	HTLV-1a-WA									HAC
007-OI	GAB	HTLV-1b						86 AA			HAC
184-NG	GAB	HTLV-1b									HAC
IYA	GAB	HTLV-1b			early STOP						ATL
12503	CAF	HTLV-1b									HAC
12504	CAF	HTLV-1b		early STOP					early STOP		HAC
12505	CAF	HTLV-1b									HAC
PH660	COD	HTLV-1b									HAC
PH1614	COD	HTLV-1b									HAC
BAK56	CMR	HTLV-1b									HAC
BAK133	CMR	HTLV-1b									HAC
BAK186	CMR	HTLV-1b									HAC
BAK40	CMR	HTLV-1b									HAC
H24	CMR	HTLV-1b									HAC
Pyl-42	CMR	HTLV-1b									HAC
Pyl-114	CMR	HTLV-1b									HAC
BAD447	CMR	HTLV-1b									HAC
HFS	NGA	HTLV-1b									HAC
2057	NGA	HTLV-1b									ATL
Nig23	NGA	HTLV-1b						99 AA			HAC
TDS	ZMB	HTLV-1b									TSP/HAM
HGS	NGA	HTLV-1b-del?						deletion of ATG		deletion of splicing site	HAC
PH1250	COD	HTLV-1b-del									HAC
PH1813	COD	HTLV-1b-del									TSP/HAM
PH1636	COD	HTLV-1b-del									TSP/HAM
418	COD	HTLV-1b-del									HAC
PH1617	RWA	HTLV-1b-del									HAC
275-HO	GAB	HTLV-1b-del									
**A.nig_Mer-Wis**	**COD**	**STLV-1b**						86AA	no ATG		
H23	CMR	HTLV-1d									HAC
358-NG	GAB	HTLV-1d							no ATG		HAC
WN-196	GAB	HTLV-1d							no ATG		HAC
057-OL	GAB	HTLV-1d							no ATG		HAC
WN-261	GAB	HTLV-1d						no ATG			HAC
WN-218	GAB	HTLV-1d									HAC
195-NG	GAB	HTLV-1f						81 AA	no ATG		HAC
BAD22	CMR	HTLV-1f						82 AA	no ATG		HAC
PH511	CAF	undefined									TSP/HAM

Notes: The different strains were analysed *in silico* and the different ORFs are presented. The sizes of the encoded P12/P8 are indicated. “No ATG” means that the start codon of the typical ORF is mutated. “Deletion of the splicing site” or “deletion of ATG” correspond to the deletion observed in HTLV-1b-del strains. “Early stop” means that an early stop codon is present in the ORFs. “Elongated ORF” means that the stop codon is mutated. Genotypes are indicated, and HTLV-1a subclades are designated as TC (Transcontinental), WA (West Africa), and NA (North Africa). Donors were either asymptomatic carriers (HAC) or patients diagnosed with Adult T-cell Leukaemia (ATL) or Tropical Spastic Paraparesis / HTLV-1 associated myelopathy (TSP/HAM).

In contrast, most HTLV-1b (non-del) strains (18/20), as well as all HTLV-1d and HTLV-1f strains, presented with altered P12/P8 encoding ORF, either due to a missing initiation codon (ATG) or the presence of premature stop codons (Table 2, Figure 4(B)). Similar truncated P12/P8 were previously reported in some HTLV-1a strains from South America [31,32] and in STLV-1 strains [33]. In addition, the absence of P13 due to a mutated start codon was frequently observed in genotypes d and f.

In HTLV-1b-del strains, the P12/P8 initiation codon is absent, as its normal location now falls within the 12-nucleotide deletion, resulting in a loss of the ORF (Figure 4(A)). This deletion also spans the splice acceptor site required for P30 expression, leading to its absence. Notably, the presence of this deletion does not appear to correlate with clinical status (Table 2).

### Looking for STLV-1 reservoirs of the different HTLV-1 genotypes and the generation of a complete genome of STLV-1 from *Allenopithecus nigroviridis*

As direct transmission of STLV-1b to humans has previously been demonstrated [10,11], we investigated whether a potential simian reservoir could be identified for the different HTLV-1 genotypes.

We compared concatenated gag-pro-pol + env + tax genes of HTLV-1 sequences with those of STLV-1 sequences obtained from African NHPs available in databases (Figure 2), the currently available complete STLV-1 genomes originate from a gorilla [34], chimpanzee [14,35], baboons [36], or African green monkeys (*Chlorocebus tantalus and C. sabeus)* [14,35,37]. Among these, the only STLV-1 strain closely related to HTLV-1 strains was characterized from a Gorilla in Cameroon (Figures 2 and 3).

We previously reported that *A. nigroviridis* originating from the COD and maintained in captivity at the *Muséum National d’Histoire Naturelle* in Paris was infected with STLV-1b strains [20]. We completely sequenced the two STLV-1b strains (which were identical) and named the sequence A.nig_Wis-Mer_COD (GenBank accession numbers PZ133440 and PZ133441). The genome is 9,047-nt long and presents a duplication of 12-bp in the pX region (position 7323–7324 in ATK-1). Analysis of accessory ORFs showed that all ORFs were present, although the P12/P8 gene encoded an 86 AA-long protein (Table 2, Figure 4(B)).

This strain belongs to the PTLV-1b genotype but forms a distinct phylogenetic branch.

Because only partial STLV-1 sequences are available in the literature, we also performed a phylogenetic analysis based on the available LTR region (593-nt) (Figure 5). This analysis indicates that the Gorilla-derived STLV-1b is the simian virus most closely related to HTLV-1b. The simian strains most closely related to HTLV-1d are found in Mandrills and *C. nictitans*, whereas viruses most closely related to HTLV-1f strains are found in Mandrills and *C. agilis*.

## Discussion

In this study, we generated the largest series of complete HTLV-1 genomes to date from Central Africa and surrounding countries, as well as from the French department of la Réunion. This work raises several important questions.

### Why is it important to study HTLV-1 from Central Africa?

Central Africa is the region of highest HTLV-1 endemicity, with several million people estimated to be infected [3]. Prevalence can reach around 15–20% among the elderly in some villages in the COD and Gabon [11,17], and this region also exhibits the highest viral diversity [6]. Despite this, HTLV-1 remains poorly studied in this area [3]. Efforts are currently underway to address this gap. For example, the first meeting specifically dedicated to HTLV-1 in Africa was recently held in Rwanda [38]. Our work actively contributes to a better understanding of the molecular epidemiology of this virus in this region and to the structuring of research groups on the subject. Except for two genotype b strains, our study provides the first available complete sequences of HTLV-1 genotypes b, d, and f.

### What advantages do full-length genomes o#er compared to short genomic fragments (e.g. LTR or env)?

HTLV-1 exhibits a low evolution rate, mainly due to its propagation by clonal expansion using mainly cellular polymerase rather than reverse transcriptase [39]. It is therefore crucial to obtain such complete sequences to encompass viral diversity.

Until now, most phylogenetic studies have been based upon partial sequence (complete or partial LTR and/or a portion of the *env* gene), leading to the definition of major genotypes based largely on phylogenetic tree topologies.

However, such fragmentary sequences make clear identification of clades and the definition of HTLV-1 variants and/or new genotypes difficult and often uncertain. Sequencing complete genomes is therefore necessary to refine our understanding of HTLV-1 variability and migration of infected populations in the area.

Interestingly, the complete sequencing of West and North African HTLV-1 strains has previously allowed us to identify the first evidence of HTLV-1 recombination [7,27]. In this study, this approach led to the identification of a novel monophyletic clade within the HTLV-1b genotype characterized by a 12-bp deletion.

We also report a divergent strain from the CAF (PH511) that does not seem to belong to any previously characterized genotype. However, we cannot ascertain whether PH511 should be considered as the prototypic strain of a novel genotype or rather grouped with the NIH00261 strain from Liberia. Indeed, no well-defined threshold of genomic divergence currently exists within HTLV-1 to determine when a new genotype designation is warranted.

The long branches observed for genotypes d and f may be artefactual, potentially resulting from sampling bias. Alternatively, they could reflect genuine differences in evolutionary rates. These viruses might circulate in certain simian species in which viral evolution proceeds more rapidly, possibly due to shorter host lifespans or more frequent inter-animal transmission (such as through biting). Such conditions would increase the number of reverse transcription rounds over a given time period – similar to the accelerated apparent evolution observed for HTLV-2 strains circulating among intravenous drug users compared with those transmitted endemically [40].

### What is the origin of the HTLV-1 viral diversity in Africa?

It is generally accepted that the presence of HTLV-1b and d-f in the human population of Central Africa results from various episodes of interspecies transmission of STLV-1 from non-human primates (NHPs) to humans.

Evidence for this zoonotic origin of these viruses is mostly based on epidemiological studies showing that a major risk factor for HTLV-1 acquisition among hunter-gatherers (compared to control groups) is being bitten by monkeys and/or apes [10,11,17,41]. The second argument is molecular/phylogenetic. Indeed, there is a great homology/similarity between HTLV-1b, d-f strains present in people bitten by or in contact with NHPs, and STLV-1 present in gorillas, chimpanzees, mandrills, *C. nictitans,* and *C*. *agilis* [42–44].

In this study, using complete sequences, we confirm that the STLV-1b strain identified in a gorilla from Cameroon shares very high similarity with human HTLV-1b from Cameroon. As some STLV-1b strains are virtually indistinguishable from human counterparts, it suggests that no adaptation to the novel human host is required.

It is unclear if direct transmission with no adaptation is still valid when discussing STLV-1 strains infecting *A. nigroviriridis* from the COD. Although these animals constitute common game in many regions, suggesting frequent opportunities for transmission to humans, complete sequence analysis reveals that this strain, while clearly belonging to genotype b, differs substantially from known HTLV-1b strains.

A thorough study generating complete STLV-1 genomes, combined with epidemiological studies, therefore represents the only viable path to identify the origin of the diverse HTLV-1 genotypes in Central Africa.

### How important is P12/P8 for HTLV-1 persistence and transmission?

While the importance of accessory proteins in viral persistence and transmission has been well reported in a macaque model, using HTLV-1a strains [45,46], their roles remain a matter of debate [30].

Indeed, some HTLV-1a strains from South America [31,32] or STLV-1 strains [33] have been reported to carry an ORF with shortened length, encoding truncated proteins of 89 amino acids rather than the typical 99 amino acids.

Here, we observed that this ORF is truncated in most genotypes except HTLV-1a [13,14,31–33].

Furthermore, we found that HTLV-1b-del strains do not encode typical P12/P8 or P30 proteins due to a 12-nt long deletion that spans the splicing acceptor of the two genes and the P12 ATG starting codon. The conserved position of this deletion across strains, along with their monophyletic clustering, strongly suggests a single ancestral deletion event. Importantly, these viruses remain infectious, as evidenced by the HTLV-1b-del strains obtained from asymptomatic carriers as well as patients with TSP/HAM or ATL, indicating that these HTLV-1b-del strains retain pathogenic potential.

In HTLV-1c strains, the presence of P12 could be offset by an alternatively spliced transcript encoding P16, which may perform analogous functions [15,16]. This p16 transcript, generated via a doubly spliced Rex-ORFI, has been recently identified in a HTLV-1a/c proviral clone. However, the deletion in HTLV-1b-del strains prevents production of this transcript. Additional studies on HTLV-1b transcripts are therefore required to determine whether alternative proteins might be expressed.

Overall, this work substantially expands the genomic landscape of HTLV-1 in Central Africa and provides a critical resource for understanding viral diversity, evolution, and the origins of human and simian PTLV-1 lineages.

## Supplementary Material

supplementary.pdf
