# Supplementary material for: HTLV-1 genetic diversity of 52 complete sequences from 14 African countries reveals novel variants and a lack of typical P12/P8 and P30 accessory proteins in HTLV-1b, d, and f genotypes
Source: Emerg Microbes Infect. 2026 Mar 25;15(1):2651463. doi: 10.1080/22221751.2026.2651463 (PMC13063333; doi:10.1080/22221751.2026.2651463)
Supplement: supplementary.pdf [file TEMI_A_2651463_SM5511.pdf]

HTLV-1 Genetic Diversity of 52 Complete Sequences from 14 African Countries Reveals Novel Variants and Lack of Canonical P12 and P30 Accessory Proteins in HTLV-1b, d and f Genotypes.

Olivier Cassar[1], Délia Doreen Djuicy[2$], Giovanni Begliomini[1$], Jill-Léa Ramassamy[1], Eldridge Fredricksen Oloumbou[3], Augustin Mouinga-Ondeme[3], Richard Njouom[2], Ambroise Marcais[4], Emilie Deruelle[4], Olivier Hermine[4], Vincente Soriano[5], Carmen de Mendoza[6], Graham Taylor[7], Philippe V. Afonso[1#], Antoine Gessain[1#]

Supplementary information

Supplementary methods

PCR Amplification and Generation of PTLV-1 Genomes

High-molecular-weight DNA was extracted from peripheral blood buffy coats using the QIAamp DNA Blood Minikit (Qiagen, Hilden, Germany) or was obtained directly from the clinical units responsible for patient management. In the laboratory, to prevent cross-contamination, all DNA extractions were performed under a biological safety cabinet in a dedicated clean room. Samples originating from related donors were systematically processed separately.

DNA samples were subjected using four overlapping PCR assays (F1-F4) covering the complete HTLV-1 genome, targeting the LTR-*gag* (F1), *gag-pro* (F2), *pol-env* (F3), and pX-LTR (F4) regions, as previously [1,2].

PCR reaction tubes were prepared in a dedicated room outside the laboratory. Each reaction had a final volume of 50 µl and contained 250 ng of DNA template; 40 µM dNTP mix (Roche, Basel, Switzerland); 5 µl of 5× Phire reaction buffer providing a final MgCl2 concentration of 1.5 mM Life Technologies, Courtaboeuf, France); 2 U of Phire hot start DNA polymerase (Life Technologies, Courtaboeuf, France); and 0.5 µM of each oligonucleotide primer (Eurofins Genomics, Germany)).

Amplification was carried out using a PIKO thermocycler (Finnzyme) under the following amplification conditions:

- Fragments(F1-F3: 98°C, 30 s; 40× (98°C, 5 s; 67°C, 5 s; 72°C, 1 min); 72°C, 1 mn
- Fragment F4: 98°C, 30 s; 40× (98°C, 5 s; 61°C, 5 s; 72°C, 1 min); 72°C, 1 min.

Hybridization temperatures were adapted for HTLV-1d and HTLV-1f strains: 69°C for F1, 68°C for F2, 67°C for F3 and 65°C for F4.

Primer locations and sequences are provided in Figure S1 and table S2.

To amplify the region spanning over the deletion in HTLV-1b-del, new primers were designed: Fo the outer PCR (492-bp): F6524_B-Delet_Out (5'-CCCTTGTTGCGCTACTCCTTCTT-3'; nt 6,545-6,567) and R7003_B-Delet_Out (5'-GAGCGGGAGAAAGAGGAGGAAAAG-3'; nt 7,013-7,036). For the inner PCR (375-bp), the primers were: F6641_B-Delet_In (5'-CCCTGTAAGCCAAGCACACAGTTA-3'; nt 6,662-6,685) and R7003_B-Delet_Out (5'-GAGCGGGAGAAAGAGGAGGAAAAG-3'; nt 7,013-7,036). The reaction mixture composition was identical to that described above. Thermocycling was performed using Piko

Thermocyler, were: 98°C for 30s; 40 cycles of 98°C for 5s, 62°C for 5s and 72°C for 20s; followed by a final extension at 72°C for 1min.

Assembly of complete PTLV-1 genomes

PCR products corresponding to positive F1-F4 sub-fragments were sent to Eurofins Genomics (Cologne, Germany) for purification and sequencing (see Figure S1, Table S2). Each sequence reaction included an aliquot of the corresponding primer (10 μM). Sequencing was performed using the Sanger method, quality check was performed by Eurofins Genomics.

Most nucleotide positions were covered by four independent sequencing reads, while a minority were covered by two reads. For positions with two reads, both were concordant and supported the same nucleotide call. For positions covered by four reads, the majority were fully concordant. In rare instances, one of the four reads was discordant, the corresponding chromatogram displayed a double peak with the minor corresponded to the nucleotide identified in the three concordant reads. In these cases, the nucleotide was assigned according to the majority rule. Using this approach, complete consensus genome sequences were generated without ambiguous positions.

Sequences are available on GenBank (see Table 1).

Complete genomes and alignment of complete genomes can be downloaded from the Figshare website: https://doi.org/10.6084/m9.figshare.31545565

[1] Cassar O, Desrames A, Marcais A, et al. Multiple recombinant events in human T-cell Leukemia virus Type 1: complete sequences of recombinant African strains. Emerg Microbes Infect. 2020;9(1):913-923.

[2] Cassar O, Einsiedel L, Afonso PV, et al. Human T-cell lymphotropic virus type 1 subtype C molecular variants among indigenous australians: new insights into the molecular epidemiology of HTLV-1 in Australo-Melanesia. PLoS Negl Trop Dis. 2013;7(9):e2418.

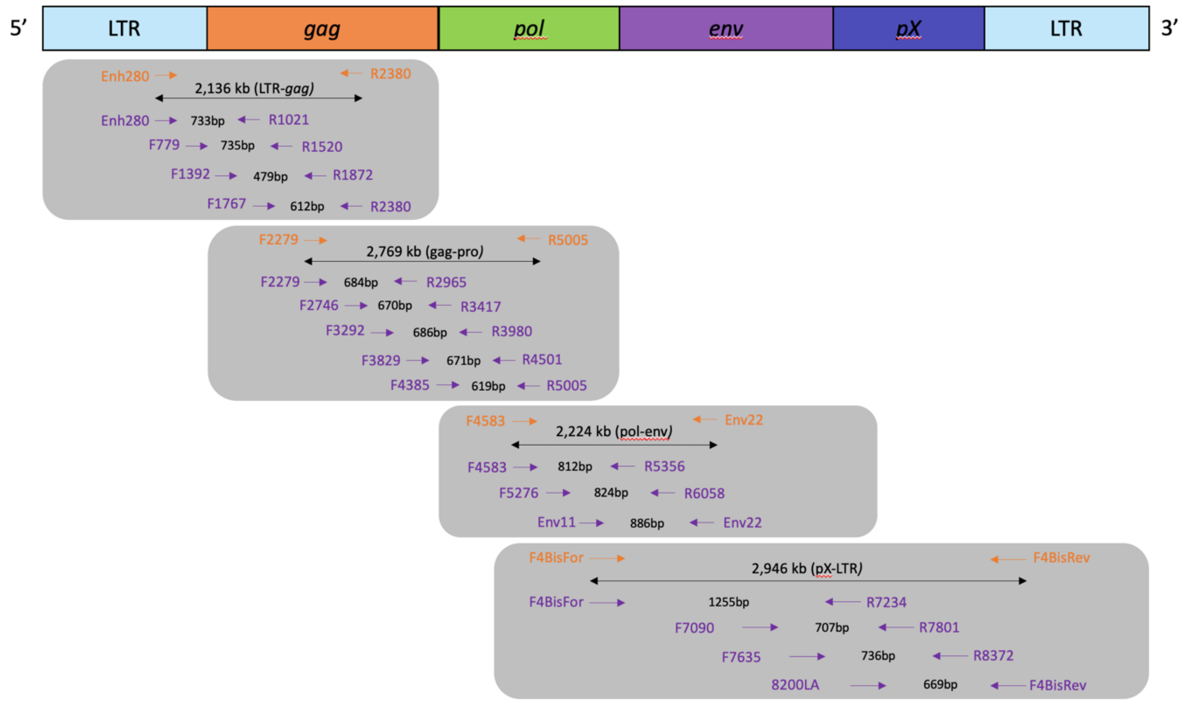

Figure S1 - PCR strategy for amplifying and sequencing complete genomes

Primers used to amplify fragments F1-4 are presented in orange. Sequences are presented in Table S1. Internal primers designed for sequencing reactions are presented in purple. Sequences are presented in table S2.

Table S1 – Sequences of the primers used for amplification.

Primer name	Sequence (5'-3')	Fragment
Enh280	TGA CGA CAA CCC CTC ACC TCA A	F1
R2380	GTC CGG AAA GGG AGG CGT ATT AG	F1
F2279	GGA GCA GAC ATG ACA GTC CTT CC	F2
R5005	GGC GGC TAT TAA GAC CAG GAA GC	F2
F4583	CAG GAG CCA TCT CAG CTA CCC	F3
Env22	GGC GAG GTG GAG TCC TTG GAG GC	F3
F4BisFor	CCC TCC TTT ACC CAT CGT TAG	F4
F4BisRev	CGC AGA ACA GAA AAC GAA AC	F4

Table S2 – Sequences of the primers used for sequencing

Primer name	Sequence (5'-3')
Enh280	TGA CGA CAA CCC CTC ACC TCA A
R1021	GGG TAT CCT TTT GGG AGT AG
F779	GTT GGG GGC TCG TCC GGG A
R1520	GTA TTC TCG CCT TAA TCC TTG
F1392	CCT CCT GCA GTA CCT TTG CTC
R1872	GCA CCG GAA GCA CGG CTG AT
F1767	AAT TAC TAC AGG CCC GAG G
R2380	GTC CGG AAA GGG AGG CGT ATT AG
F2279	GGA GCA GAC ATG ACA GTC CTT CC
R2965	TTT AAA CCC TTG GGG TAG
F2746	CCT GGC GAT TCA TCC ACG ACC
R3417	TAA TGA TTG AAC TTG AGA AGG A
F3292	TCC CGC TGG GCG CTA CCT GAA C
R3980	AAG GGG GAA TGA TCT TTG TGA
F3829	CTG GAG AAC TTT GGA ACA CT
R4501	TAT ATT TGA AAT GGG TAA TG
F4385	TGC AAG GGG CAA CCA CAA CTG
R5005	GGC GGC TAT TAA GAC CAG GAA GC
F4583	CAG GAG CCA TCT CAG CTA CCC
R5356	TGA TCT GCT GAA AGG GCC AG
F5276	ATT ACA GCC CCA GCT GCT GTA C
R6058	CAG GAT GAG GGA GTT ATG ACA
Env11	TGG CAC GTC CTG TAC TCT CCC AAC
Env22	GGC GAG GTG GAG TCC TTG GAG GC
F4BisFor	CCC TCC TTT ACC CAT CGT TAG
R7234	CGG AGG ACC TGC TGG TGG AGG A
F7090	TCA CGA TGC GTT TCC CCG CGA G
R7801	GCA GGA GGG GCC AGG TGA TG
F7635	CAA CAT TCC ACC CTC CTT CCT CCA
R8372	CTG GGC CCT GAC CTT TTC AGA C
F8200LA	CTC ACA CGG CCT CAT ACA GTA CTC
F4BisRev	CGC AGA ACA GAA AAC GAA AC

Supplementary figures

Figure S2 – Phylogenetic tree based on the alignment of concatenated gag-pro-pol+env+tax genes / PhyML in IQ-Tree

Phylogenetic analysis was performed using a 6,915-bp alignment of concatenated gag-pro-pol+env+tax genes. The tree was inferred with PhyML and IQ-TREE under the HKY+I+G selection model.

Branch lengths are drawn to scale, with the bar indicating 0.01 nucleotide replacement per site. Numbers at each node indicate the boot strap values obtained using the ultrafast bootstrap method (n=1,000).

The newly generated sequences are presented in red for HTLV-1. STLV-1 strains are indicated in blue with the first letters referring to the non-human primate (NHP) species.

Countries are indicated by their three-letter code (ISO 3166).

TC, Transcontinental; WA, West African; PTLV, and HTLV mean primate and human T-lymphotropic virus, respectively.

Figure S3 – Phylogenetic tree based on the alignment of concatenated gag-pro-pol+env+tax genes / Parsimony

Phylogenetic analysis was performed using a 6,915-bp alignment of concatenated gag-pro-pol+env+tax genes, using a maximum parsimony approach implemented in SeaView. The tree was inferred from 5 randomization replicates.

Branch lengths are drawn to scale, with the bar indicating 0.005 nucleotide replacement per site. Numbers on each node indicate the boot strap-values (percentages) obtained using the ultrafast bootstrap method (n=100). HTLV-1c strains are used as an outgroup.

The newly obtained sequences are presented in red for HTLV-1. STLV-1 strains are indicated in blue, with the first letters correspond to the NHP species.

Countries are indicated using their three-letter codes (ISO 3166).

TC, Transcontinental; JPN, Japan; PTLV, and HTLV mean primate and human T-lymphotropic virus, respectively.

Figure S4 – Phylogenetic tree based on the alignment without ambiguous sites from the LTR alignment.

Phylogenetic analysis was performed using a 746-bp alignment of non-ambiguous LTR sites, using PhyML implemented in Seaview under the GTR+I+G substitution model.
Branch lengths are drawn to scale, with the bar indicating 0.01 nucleotide replacement per site.
Numbers at each node indicate aLRT support values (probabilities).
The newly generated sequences are shown in red for HTLV-1. STLV-1 strains are presented in blue, and the first letters correspond to the NHP species.
Countries are indicated using their three-letter codes (ISO 3166).
PTLV, and HTLV mean primate and human T-lymphotropic virus, respectively.

Figure S2

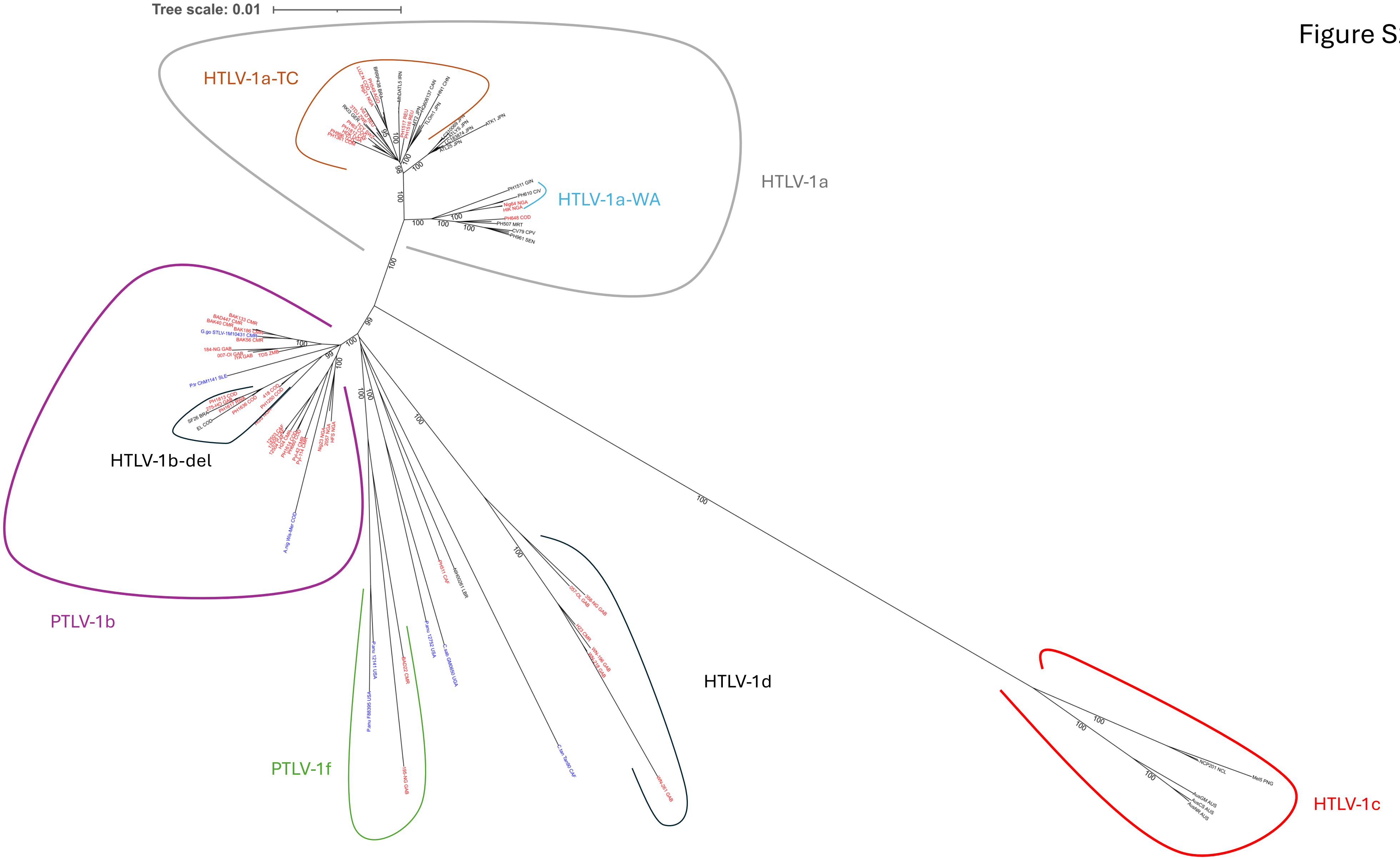
Tree scale: 0.01
HTLV-1a-TC
HTLV-1a
HTLV-1a-WA
PTLV-1b
HTLV-1b-del
PTLV-1f
HTLV-1d
HTLV-1c

Figure S3

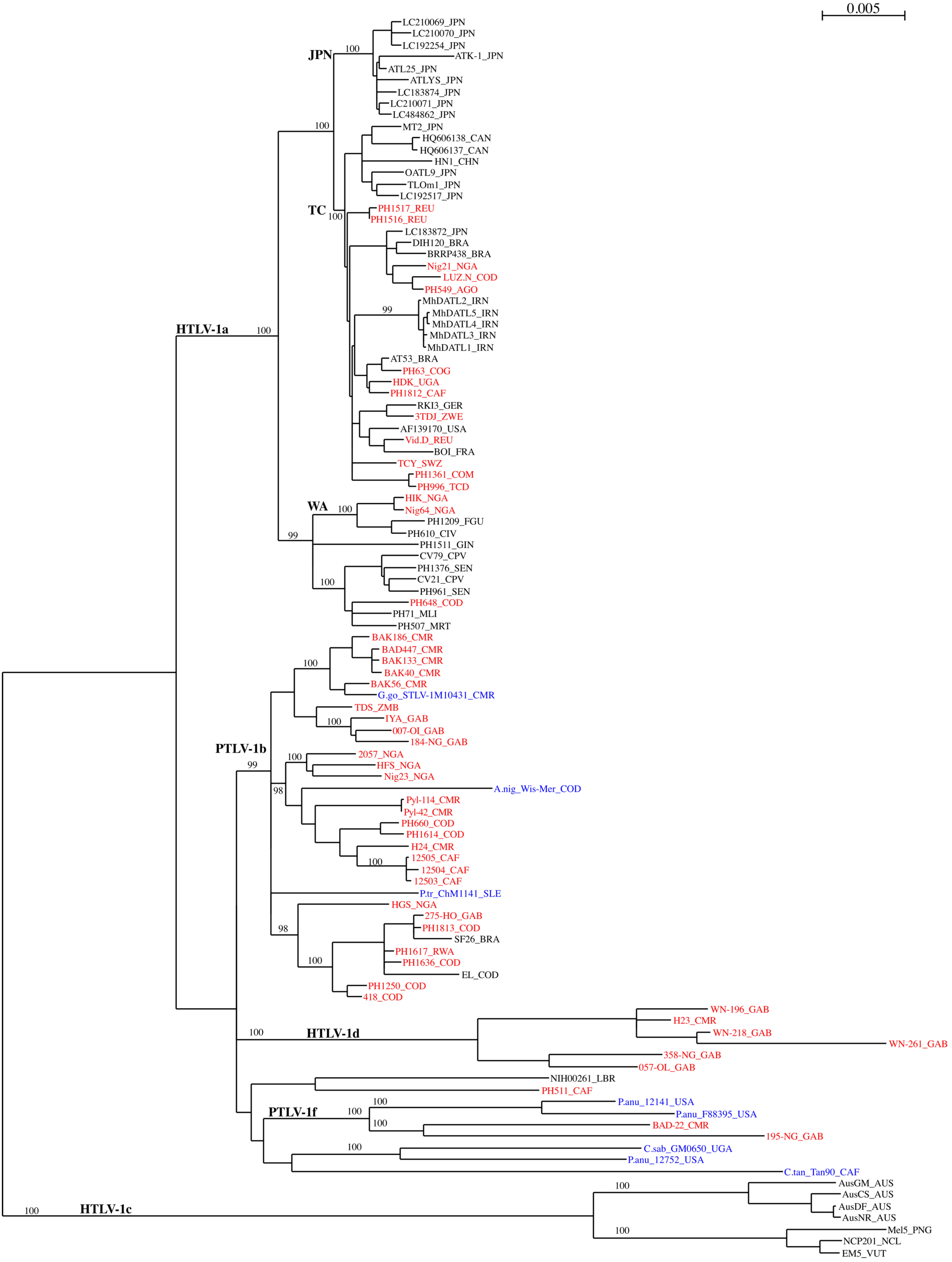
0.005
JPN
TC
WA
HTLV-1a
PTLV-1b
HTLV-1d
PTLV-1f
HTLV-1c
LC210069_JPN
LC210070_JPN
LC192254_JPN
ATK-1_JPN
ATL25_JPN
ATLYS_JPN
LC183874_JPN
LC210071_JPN
LC484862_JPN
MT2_JPN
HQ606138_CAN
HQ606137_CAN
HN1_CHN
OATL9_JPN
TLOm1_JPN
LC192517_JPN
PH1517_REU
PH1516_REU
LC183872_JPN
DIH120_BRA
BRRP438_BRA
Nig21_NGA
LUZ.N_COD
PH549_AGO
MhDATL2_IRN
MhDATL5_IRN
MhDATL4_IRN
MhDATL3_IRN
MhDATL1_IRN
AT53_BRA
PH63_COG
HDK_UGA
PH1812_CAF
RKI3_GER
3TDJ_ZWE
AF139170_USA
Vid.D_REU
BOI_FRA
TCY_SWZ
PH1361_COM
PH996_TCD
HIK_NGA
Nig64_NGA
PH1209_FGU
PH610_CIV
PH1511_GIN
CV79_CPV
PH1376_SEN
CV21_CPV
PH961_SEN
PH648_COD
PH71_MLI
PH507_MRT
BAK186_CMR
BAD447_CMR
BAK133_CMR
BAK40_CMR
BAK56_CMR
G.go_STLV-1M10431_CMR
TDS_ZMB
IYA_GAB
007-OI_GAB
184-NG_GAB
2057_NGA
HFS_NGA
Nig23_NGA
A.nig_Wis-Mer_COD
Pyl-114_CMR
Pyl-42_CMR
PH660_COD
PH1614_COD
H24_CMR
12505_CAF
12504_CAF
12503_CAF
P.tr_ChM1141_SLE
HGS_NGA
275-HO_GAB
PH1813_COD
SF26_BRA
PH1617_RWA
PH1636_COD
EL_COD
PH1250_COD
418_COD
WN-196_GAB
H23_CMR
WN-218_GAB
WN-261_GAB
358-NG_GAB
057-OL_GAB
NIH00261_LBR
PH511_CAF
P.anu_12141_USA
P.anu_F88395_USA
BAD-22_CMR
195-NG_GAB
C.sab_GM0650_UGA
P.anu_12752_USA
C.tan_Tan90_CAF
AusGM_AUS
AusCS_AUS
AusDF_AUS
AusNR_AUS
Mel5_PNG
NCP201_NCL
EM5_VUT

Figure S4

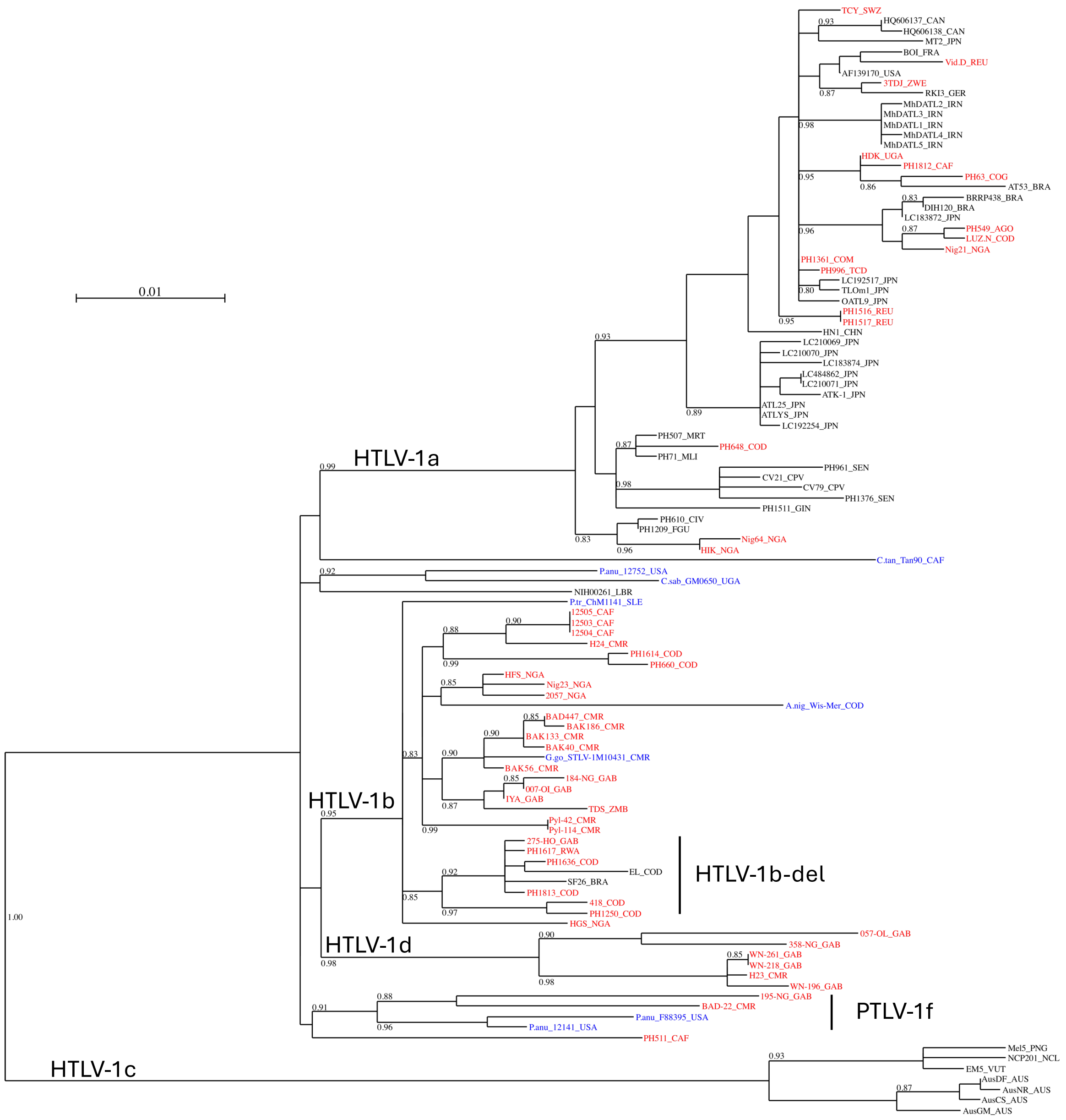

0.01
TCY_SWZ
0.93
HQ606137_CAN
HQ606138_CAN
MT2_JPN
BOI_FRA
Vid.D_REU
AF139170_USA
3TDJ_ZWE
RKI3_GER
0.87
MhDATL2_IRN
MhDATL3_IRN
MhDATL1_IRN
MhDATL4_IRN
MhDATL5_IRN
0.98
HDK_UGA
PH1812_CAF
PH63_COG
AT53_BRA
0.95
0.86
0.83
BRRP438_BRA
DIH120_BRA
LC183872_JPN
0.87
PH549_AGO
LUZ.N_COD
Nig21_NGA
0.96
PH1361_COM
PH996_TCD
LC192517_JPN
TLOm1_JPN
0.80
OATL9_JPN
PH1516_REU
PH1517_REU
0.95
HN1_CHN
0.93
LC210069_JPN
LC210070_JPN
LC183874_JPN
LC484862_JPN
LC210071_JPN
ATK-1_JPN
ATL25_JPN
ATLYS_JPN
LC192254_JPN
0.89
PH507_MRT
0.87
PH648_COD
PH71_MLI
HTLV-1a
0.99
PH961_SEN
CV21_CPV
CV79_CPV
0.98
PH1376_SEN
PH1511_GIN
PH610_CIV
PH1209_FGU
0.83
Nig64_NGA
HIK_NGA
0.96
C.tan_Tan90_CAF
0.92
P.anu_12752_USA
C.sab_GM0650_UGA
NIH00261_LBR
P.tr_ChM1141_SLE
12505_CAF
0.90
12503_CAF
12504_CAF
0.88
H24_CMR
PH1614_COD
0.99
PH660_COD
HFS_NGA
0.85
Nig23_NGA
2057_NGA
A.nig_Wis-Mer_COD
0.85
BAD447_CMR
BAK186_CMR
0.90
BAK133_CMR
BAK40_CMR
0.83
0.90
G.go_STLV-1M10431_CMR
BAK56_CMR
0.85
184-NG_GAB
007-OI_GAB
IYA_GAB
0.87
TDS_ZMB
HTLV-1b
0.95
Pyl-42_CMR
Pyl-114_CMR
0.99
275-HO_GAB
PH1617_RWA
PH1636_COD
EL_COD
0.92
SF26_BRA
HTLV-1b-del
PH1813_COD
0.85
418_COD
0.97
PH1250_COD
HGS_NGA
1.00
0.90
057-OL_GAB
358-NG_GAB
HTLV-1d
0.98
0.85
WN-261_GAB
WN-218_GAB
H23_CMR
0.98
WN-196_GAB
0.88
195-NG_GAB
0.91
BAD-22_CMR
PTLV-1f
P.anu_F88395_USA
0.96
P.anu_12141_USA
PH511_CAF
Mel5_PNG
0.93
NCP201_NCL
EM5_VUT
HTLV-1c
AusDF_AUS
0.87
AusNR_AUS
AusCS_AUS
AusGM_AUS
